# Managing multi-functional peri-urban landscapes: Impacts of horse-keeping on water quality

**DOI:** 10.1007/s13280-023-01955-9

**Published:** 2023-11-16

**Authors:** Linda Kumblad, Mona Petersson, Helena Aronsson, Patrik Dinnétz, Lisbet Norberg, Camilla Winqvist, Emil Rydin, Monica Hammer

**Affiliations:** 1 Baltic Sea Center, Stockholm University, 106 91 Stockholm, Sweden; 2 School of Natural Science, Technology, and Environmental Studies, Department of Sustainability, Environment, and Global Development, Södertörn University, 141 89 Huddinge, Sweden; 3 Department of Soil and Environment, Swedish University of Agricultural Sciences, Box 7014, 750 07 Uppsala, Sweden; 4 Rejlers Sverige AB, Stationsgatan 12, 753 40 Uppsala, Sweden

**Keywords:** Eutrophication, Land use modelling, Local measures, Nutrient load assessment, Water runoff modelling

## Abstract

Eutrophication assessments in water management to quantify nutrient loads and identify mitigating measures seldom include the contribution from horse facilities. This may be due to lack of appropriate methods, limited resources, or the belief that the impact from horses is insignificant. However, the recreational horse sector is growing, predominantly in multi-functional peri-urban landscapes. We applied an ecosystem management approach to quantify nutrient loads from horse facilities in the Stockholm Region, Sweden. We found that horses increased the total loads with 30–40% P and 20–45% N, with average area-specific loads of 1.2 kg P and 7.6 kg N ha^−1^ year^−1^. Identified local risk factors included manure management practices, trampling severity, soil condition and closeness to water. Comparisons of assessment methods showed that literature standard values of area-specific loads and water runoff may be sufficient at the catchment level, but in small and more complex catchments, measurements and local knowledge are needed.

## Introduction

Eutrophication due to excessive inputs of nitrogen (N) and phosphorus (P) is one of the most serious environmental threats for many lakes and coastal areas, including Lake Mälaren (Sweden) and the Baltic Sea (Boesch et al. 2006; Elmgren et al. 2015; HELCOM 2018a, b; Drakare et al. 2022; Vigouroux and Destouni 2022; VISS 2022). The nutrients originate from a range of different sources in different types of land use, and are often higher in areas with much arable land and high animal density (Hong et al. 2012, 2017; Svanbäck et al. 2019). Common nutrient abatement measures to mitigate eutrophication are improved urban wastewater treatment, improved manure management and reduction in fertilizer application in agriculture (Hong et al. 2017; McCrackin et al. 2018a; Andersson et al. 2022).

Mitigation measures according to the Water Framework Directive should involve stakeholders and be based on a catchment perspective (EC 2000). Hence, measures must be adapted to local circumstances, where changes in land use may require the development of local capacity building and management strategies. One important nutrient source is the peri-urban transition zone characterized by a diverse and fragmented land use and an increasing demand for recreational ecosystem services (Antrop 2004; Orsini 2013). In the peri-urban areas, agriculture for food production is replaced with recreational land use, including allotment gardens, golf courses and equestrian facilities (Bomans et al. 2011; Elgåker 2012; Zasada et al. 2013; Plieninger et al. 2016).

In this study, we focus on the impact from horse facilities on water quality. The horse sector in European and North American countries is growing. In EU, the number of equestrians increases with approximately 5% per year, and currently, there are at least 6 million hobby and sport horses in Europe (European Horse Network 2023). In Sweden, the number of horses exceeds the number of dairy cows (Swedish Board of Agriculture 2022). Around 76% of the approximately 350 000 horses are kept in peri-urban and urban environments (Swedish Board of Agriculture 2017). In contrast to livestock management, the nutrient load from horse activities has only been included in management plans and action programmes in Sweden to a very limited degree, but studies indicate that nutrient leakage from horse paddocks can have negative impact on water quality (Airaksinen et al. 2007; Parvage et al. 2011). There are management differences between livestock husbandry and horse-keeping that may affect nutrient load (Hammer et al. 2017). Cattle are grazing in pastures during the vegetation period but are usually kept indoors during the winter. According to Swedish animal protection regulations, horses must spend at least 1 h outdoors in a paddock where they can move freely each day year around. This increases the risk of over-grazing, extensive trampling damages and the deposition of horse manure and urine in paddocks also during winter (Hammer et al. 2020; Viksten et al. 2016). Also, horses are present in the surrounding landscape to a higher degree than cattle, since horseback riding along bridleways is an important part of recreational horse-keeping.

Accumulated anthropogenic P in the landscape remains mobile for decades and contribute to eutrophication of surface waters (McCrackin et al. 2018b). Horse manure that is not regularly collected from paddocks increases the risk of P losses from manure heaps to soil and water (Airaksinen et al. 2007; Aronsson et al. 2022). Also, the long-term accumulation of P in the soil of intensively used paddocks, especially in areas for feeding and defecation (Airaksinen et al. 2007; Parvage et al. 2013), will contribute to increased risk of P losses due to the strong relationship between increasing soil P content (soil P saturation) and the amount of water-soluble P in the soil (Heckrath et al. 1995; Börling et al. 2004). Thus, a high density of horses on land formerly used as arable fields or for cattle grazing probably increases nutrient loads to adjacent waters, through surface runoff, or drain tiles, causing eutrophication (Airaksinen et al. 2007; Hammer et al. 2017; Parvage et al. 2011, 2013).

To design and target effective mitigation measures and encourage local mitigation activities, there is a need of field data and local knowledge of actual nutrient losses, both for individual paddocks, as well as on farm and catchment levels. This includes management and mitigation practices regarding paddocks and manure management at the farm level as well as an understanding of the role of farm location in the landscape and catchment.

In this study, we apply an ecosystem management perspective to transforming land use patterns in multi-functional peri-urban landscapes, focusing on the effects of increased horse-keeping on nutrient loads and water quality. The aims were to investigate if equine facilities contribute to nutrient loads to adjacent surface waters, identify the risk factors for nutrient leakage and to identify, test and evaluate a suitable method to quantify nutrient loads from horse-keeping in the context of other diffuse and point sources in peri-urban landscapes. To do this, we used calculations based on literature standard values as well as field measurements and water runoff modelling for sub-catchments including horse activities on five horse farms located in four different catchment areas in the Stockholm Region, Sweden.

## Materials and methods

### Study area

**Fig. 1:**
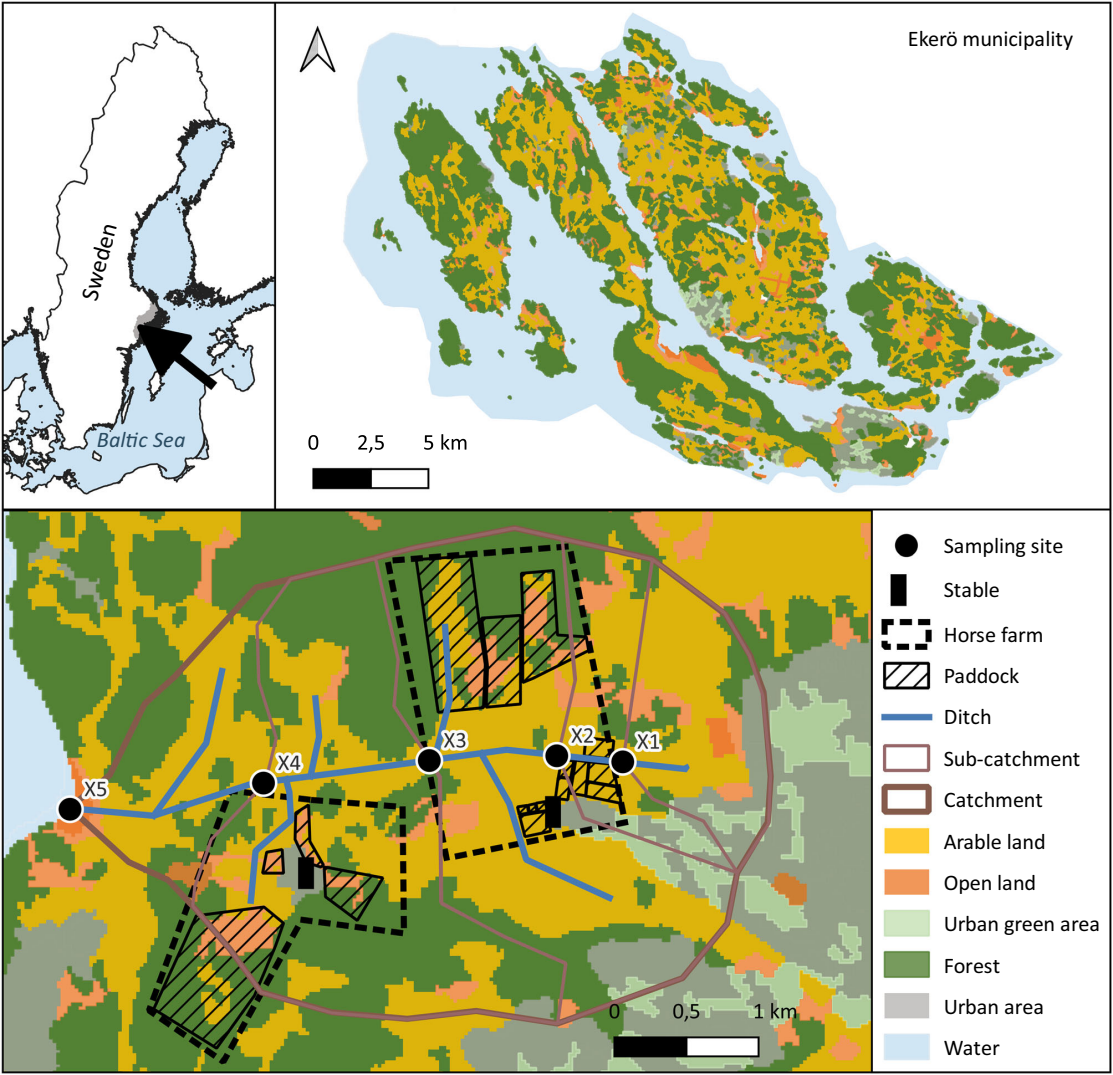
Location of Stockholm region and Ekerö municipality with a schematic illustration of a catchment divided into sub-catchments for each sampling site. To keep the farms anonymous the name of the sampling site is fictive and just illustrates the study design. Landcover data downloaded from the Swedish Environmental Protection Agency (open data)

The study was performed in Ekerö municipality, 10 km from Stockholm city centre (Fig. 1). Ekerö has ca 29 000 inhabitants and is one of the most horse-dense municipalities in Sweden with ca 2000 horses in around 130 facilities, predominantly for recreational horse-keeping (Ekerö municipality 2018). The municipality is recognized for its high natural and cultural values and is comprised of an archipelago in Lake Mälaren (57% land, 43% water), which discharges into the heavily eutrophicated coastal areas of the Baltic Proper (Walve et al. 2018). Lake Mälaren is an important public water supply for the Stockholm region, and currently, the ecological status is good to moderate (VISS 2022). The land use (13% urban area, 43% forest, 30% arable land, 14% open land) follows the soil distribution where soils in the upper part of the terrain are forested, and arable land is located in the lower areas where clay soils dominate (Fig. 1). Small catchments dominate the runoff, drained by ground water and small ditches and streams. Most of the arable land is artificially tile drained. In the cold temperate climate, the runoff is normally highest in the winter with a peak in the early spring (February–March) and lowest in the summer during the vegetation period (July–August).

### Horse facilities and sampling sites

Five horse facilities in four water catchments (A–D) were identified via map analyses and field visits. In total, 18 sampling sites for water were selected. The sampling sites represent sub-catchments “upstream”, “at/close to”, or “downstream” horse facilities, or a reference area with only forest. For each water sampling site, the sub-catchment size and the area used for different land use were calculated, and the runoff was measured and modelled. Paddocks in the vicinity of sampling sites were monitored by visual observations year-round and the status of the vegetation cover, trampling and waterlogging was documented. We use the word paddock for fenced areas of varying sizes, where horses, alone or in smaller groups, spend parts of the day all year-round. The grass cover in paddocks varied between fully covered to bare. Grazing areas refer to larger pastures, mainly used for summer grazing (June–August), where the horses can spend a few weeks to months feeding on the grass.

All five horse facilities included in the study are livery yards for private horses, averaging ca 35 horses per facility and horse density of ca 4.5 horses ha^−1^, but varied substantially between the facilities and over the year (Table 1). Information was gathered about the horse-keeping practices including feeding regimes, daily and seasonal turn-out routines in paddocks and summer grazing areas, as well as the manure management, mucking regimes, overflow events and ditch-cleaning activities that have taken place. The horses are mainly fed in the stables with combinations of roughage and different concentrates, minerals and vitamins, usually adapted to the individual horse and level of activity. The pasture in the paddocks is a minor supplement, except for summer grazing periods. The horse facilities represent different settings of pastures, paddocks and stable facilities, mixed with other land use in their respective catchment areas.

**Table 1:** Surface area of catchment, area for paddocks and grazing, number of horses and horse densities in the studied water catchments

Water catchment	Catchment area (ha)	Area for paddock and grazing [ha (%)]	Number of horses (#)	Horse density (#/ha)
A	52	14 (28%)	30	2.1
B	81	6 (7%)	30	5.0
C	74	13 (18%)	45	3.4
D	21	3 (16%)	25	7.6
Average	57	9 (16%)	33	4.5

### Phosphorus accumulation in the soil

To assess the risk of P leaching from paddocks, P accumulation in the soil of horse paddocks was studied in one of the catchments (A). Two adjacent horse paddocks (ca 2.6 horses ha^−1^) with 30–50 years of horse-keeping, and a grazing area used for 2 years were sampled. The clay contents of the soils were 27–39%. An adjacent arable field (54% clay) and a nearby forest soil were used as reference areas. The paddocks had a poor or no (near the entrance) vegetation cover at sampling while the grazing area had intact grass cover all year around.

Samples from different parts of the grazing areas were taken in September (0–10 cm depth) and in November (0–10, 10–20 and 20–30 cm depth) 2020 as composites sample consisting of 8 sub-samples.

The soil was analysed for total nitrogen (N), total phosphorus (P), total carbon (C), ammonium-lactate soluble P (P-AL), hereafter referred to as easily soluble P, and CaCl_2_-soluble P, a proxy for P directly available for leaching. Also, ammonium-lactate soluble iron (Fe) and aluminium (Al) were analysed to estimate the degree of P saturation of the soil (DPS), calculated according to Ulén (2006). Total P and P-AL, Fe, and Al were analysed with ICP, the later after extraction with ammonium-lactate at pH 3.75 (Egnér et al. 1960). Total N and C were analysed with Leco. For estimation of directly leachable P (Blombäck et al. 2021), soil samples were extracted with CaCl_2_ (0.01 M) and analysed colorimetrically (Murphy and Riley 1962).

### Identification of land use, catchment areas and water runoff

**Fig. 2:**
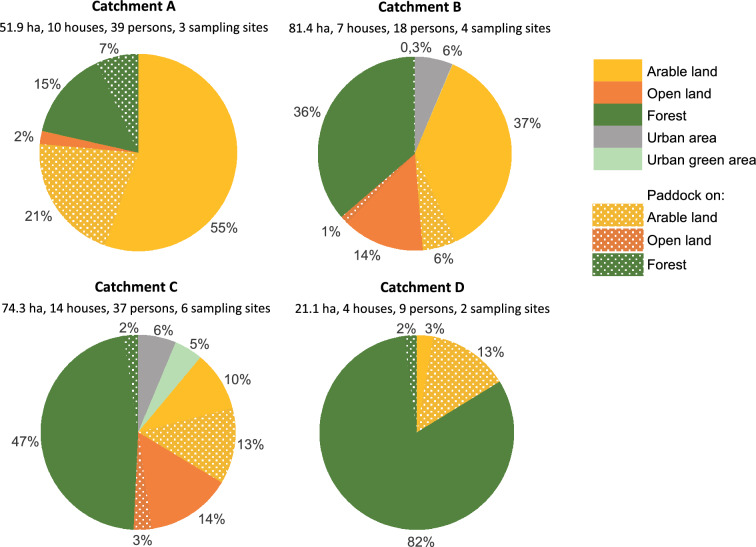
Surface area (ha), number of houses and persons, sampling sites and land use distribution of the catchments of the horse farms, including paddocks and assumed land use if they were not used as paddocks

Catchment areas, flow direction and flow accumulation were identified using altitude data from Lantmäteriet (Swedish mapping authority) and hydrological calculation models available in ArcGIS Pro 2.5. Delimitation of water divides for sub-catchments was done manually based on the GIS-model result (Fig. 1). Six different land use classes (urban area, urban green area, arable land, pasture, open land and forest) were identified and extracted from CORINE landcover data from 2012. The paddocks and grazing areas for each horse facility, as well as the number of houses with private sewers were mapped using aerial photos from 2019 (Lantmäteriet) (Fig. 2).

#### The runoff model

The runoff was calculated using the HBV model described in Bergström (1992) and Lindström et al. (1996) based on the general water balance of precipitation, evapotranspiration, runoff, and storage. No lakes were present in the studied catchment areas. The model divides the catchment area into sub-catchments and the input data were daily temperature and precipitation, which were downloaded from the closest weather station (SMHI open data). The potential evapotranspiration was calculated using the average daily temperature, and the sun inclination for the day of the year according to Penman (1948). To estimate the actual evapotranspiration, an interception routine was applied described in Lindström et al. 1996). The water storage capacity of the forest canopy was set to 2 mm, and the storage capacity of open land was set to 0 mm.

Each sub-catchment was divided into two separate model units, one for forest (sandy glacial till), and the other for open land (glacial/post-glacial clay) based on landcover, and soil type, e.g. parent material (SGU open data). Each model units contributing separately to the sub-catchment runoff. The field capacity was set to 244 mm for the till, and 366 mm for the clay based on a study by Rodhe et al. (2006). The idea was to keep the runoff model simple using parameters available from studies with similar conditions (region and climate). The pros and cons of the simple setup of the HBV model are discussed in relation to other hydrological models in a paper by Seibert and Bergström (2022). A weakness of the model was the possibility to calibrate the surface runoff continuously during the measurement period in the previously ungauged catchments. Two field methods were used depending on the site condition, the float method (velocity-area principle) and the volume per time method. The float method measures the surface velocity and was re-calculated to mean velocity for the channel section using a coefficient (e.g. 0.8), then multiplied with the area of the cross-section (Davids et al. 2019). The volume per time method was used for sites where the cross-section allowed collecting the total runoff into a container, measuring the time to fill the known volume, and no further calculations were needed. Field measurements were scattered during the sampling period in relation to occasions with precipitation, occasionally sampling was not possible at all sites when ditches were partly frozen and not reachable, or dry. Another problem was human impact such as ditch cleaning.

To validate the model, the modelled runoff was compared with the estimated runoff for the two regions, where the field sites were situated (SMHI open data, SVAR_2016_3). The SVAR estimation for the studied period was 160 mm and 161 mm. The model values for water runoff varied between ca 85 mm in areas dominated by forest, up to ca 135 mm in areas with more arable land. Both the SVAR estimation and the model had approximately 30% drier conditions during the studied period in relation to the 30-year average. The validation suggests a slight underestimation by the runoff model.

### Two approaches to estimate the nutrient load from catchments with horse facilities

We used two different approaches to estimate the nutrient loads, with the attempt to compare and evaluate the possible quantification methods for water quality assessment in areas with a high density of horses (Table 2).

**Table 2:**
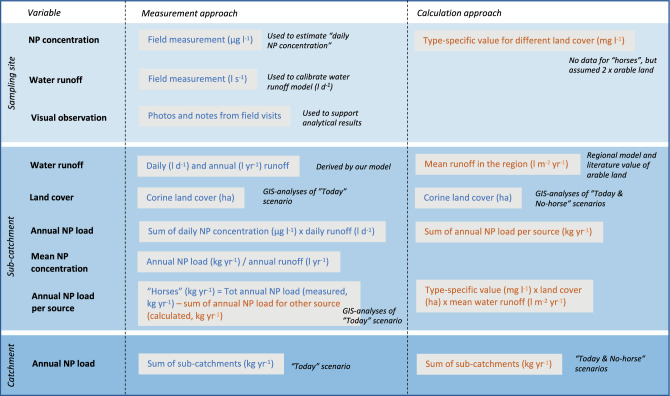
Summary of how variables were derived in the “Measurement” and the “Calculation” approach, respectively, at the three different levels of scale; sampling site, sub-catchment and catchment. NP stands for nutrient, i.e. total and dissolved N and P. Blue text represents data collected or derived from site-specific measurements and modelling, whereas orange text represents literature data

#### The “Calculation” approach

Nutrient loads estimated with the “Calculation” approach were based on standard values on area-specific load (kg ha^−1^ year^−1^) derived from type-specific nutrient concentrations (mg l^−1^) for land use types (Table 3), the runoff (mm year^−1^) from the respective sub-catchment and the proportion of respective land use (%) (Table 2). The load from paddocks was assumed to be twice the load for arable land based on the findings by Parvage et al. (2011), where the mean concentration of P in the paddock was three times higher than the arable land, and the easily soluble P in the soil was close to two times higher in the paddock. The contribution from small sewers was estimated from type and age of respective sewage solution, the number of persons per household, literature values on nutrient content in the wastewater (Jönsson et al. 2005), reduction effectiveness of sewage solutions (Palm et al. 2002; Hübinette 2009) and an assumed nutrient retention (30%) between the outlet from the respective sewage system and the recipient water (Hansson et al. 2019). To investigate the impact of horse facilities on the nutrient load, two scenarios were compared for the calculation approach: the “today-scenario” and the “no-horse-scenario”, where an alternate land use to paddocks was assumed based on the type of soil and present land use in the non-horse areas with similar conditions (Fig. 2).

**Table 3:** Standard values used for type-specific nutrient concentration (mg l^−1^) for total P (TP) and total N (TN) load for different land uses

	Type-specific concentration		References
	mg TP l^−1^	mg TN l^−1^	
Forest	0.013	0.52	Ejhed et al. (2016), Hansson et al. (2019)
Open land	0.026	1.50	Ejhed et al. (2016)
Arable land	0.280	3.00	SMED (2019)
Paddock			Assumption “two times arable land”
Urban area	0.135	1.67	StormTac (2021)
Urban green area	0.140	1.05	StormTac (2021)

#### The “Field measurement” approach

Nutrient load estimates from “Field measurements” were based on empirical data on nutrient concentrations and modelled water runoff (Table 2). Water samples were collected approximately every 2 to 3 weeks from March 2020 to March 2021 (in total 18 occasions), at periods of sufficient water flow for sampling. During high runoff, the sampling was more frequent. The water samples were analysed for total phosphorus (P) and nitrogen (N), as well as phosphate P, ammonium N and nitrate–nitrite N, hereafter referred to as dissolved P and N, respectively. All water samples were analysed at Erken Research Laboratory at Uppsala University using spectrophotometrically methods (SIS 1996, 2004, 2005). The water samples were kept dark on cooler blocks in a cooler box (aiming at 2–8 °C) from sampling to analysis that was performed the same day.

The annual total P and N loads were calculated as the sum of the daily loads. Daily loads were derived from an interpolation between the 18 field measurements with local polynomial regression fitting using the loess function in R 4.0.5 (R Core Team 2021) with span 0.75, polynomial degree = 2, and Gaussian residual distribution. The annual mean concentration of total P and N were calculated as the ratio of the annual total P and N load and the annual runoff (Table 2). To investigate the potential sources of the measured nutrient loads, we used the estimates from the “Calculation” approach, and nutrient load from paddocks was assumed to be the residual load of the total load and the sum of other sources.

### Statistical analyses

Differences in element contents of the soil (0–10 cm depth) in the three paddocks at the first sampling, and differences between different soil depths at the second sampling, were tested with one-way ANOVA (*p* < 0.05). Tukey’s post hoc test was used for examining the pairwise differences between depths. Associations among variables were tested with regression analysis. All statistical analyses on soil data were performed with SAS JMP Pro 15.

Measurements of environmental N and P load were analysed as functions of the different land use types present in each sub-catchment with linear mixed models using package lme4 (Bates et al. 2015) in R 4.0.5 (R Core Team 2021). Log total P or N load, mean total P or N concentration, dissolved P or N concentration and square root of total P or N load per hectare were analysed individually as response variables in eight different models. For all models, we included the total sub-catchment area, location of sampling site within the farm, nutrient load from private sewers and the different land use types estimated as their proportion of the total sub-catchment area as fixed factors. We sampled at several locations within each farm; therefore, all models also included farm as a random factor to control for the correlative structure within farms. All full models included all fixed and all random factors (Eq. 1, example of full model). We used *p* values and AIC values for a backwards selection procedure to select the simplest most informative final model (Eq. 2, example of final model). A non-significant variable dropped was considered uninformative if it did not conflict with the change in AIC and instead resulted in a less informative model with fewer significant explanatory variables.

1 $${\text{log total P load }}\sim {\text{ Sub-catchment Area }} + {\text{ Location}}^{{1}} + {\text{ P load from Sewers }} + {\text{ Paddock}}^{{2}} + {\text{ Arable land}}^{{2}} + {\text{ Open land}}^{{2}} + {\text{ Forest}}^{{2}} + {\text{ Urban area}}^{{2}} + {\text{ Urban green area}}^{{2}} + \, \left( {{1}|{\text{Farm}}} \right)^{{3}}$$

where ^1^Location around horse facility [upstream, at/close to, downstream], ^2^Proportion of the total sub-catchment area, ^3^Random factor treating all measurements from the same farm as dependent.

2 $${\text{log total P load }}\sim {\text{ Location }} + {\text{ P load from Sewers }} + {\text{ Paddock }} + {\text{ Open land }} + \, \left( {{1}|{\text{Farm}}} \right)$$

## Results

The presentation of the results is organized in an increasing order of scale and complexity, starting with the results from the field measurements of P in soil and N and P in ditch waters in the 18 sampling sites. Based on the two methods described in the methods section, estimates are then presented for nutrient load at the sub-catchment-level, as well as at the catchment level, including the influence of the horse facilities and other land uses.

### Phosphorus accumulation in the soil and related risk of P losses

**Fig. 3:**
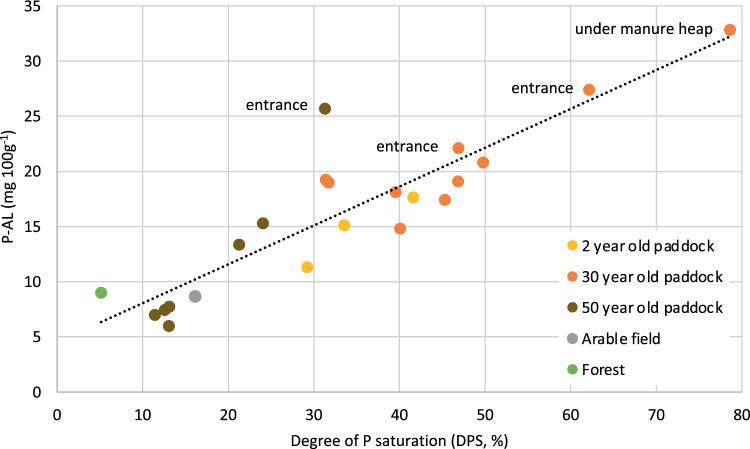
Relationship between ammonium-lactate soluble phosphorus in the soil (P-AL) in paddock, arable field and forest and degree of phosphorus saturation (DPS) (*y* = 4.5 + 0.35*x*, *R*^2^ = 0.8, *p* < 0.0001). Three of the soil samples were collected at entrances of paddocks and one under a manure heap in a paddock

There was an accumulation of organic matter and nutrients close to the soil surface of the paddocks (0–10 cm depth), compared to soil further down (10–30 cm depth). The accumulation in the top 10 cm was significant for total N and C and P-CaCl_2_, but not for P and P-AL (Table 4). In the samples taken at 0–10 cm depth, there was a strong significant positive relationship between the pool of easily soluble P (P-AL) and the degree of soil P saturation (DPS) (Fig. 3), especially at entrances and in defecation places. In these areas, and at the entrance of the 50-year-old paddock, P directly available for leaching (P-CaCl_2_) constituted 0.1–0.5% of the easily soluble pool of P at 0–10 cm depth. Despite the long history of horse-keeping in the 50-year-old paddock, soluble P and DPS were lower than in the other paddocks (Fig. 3).

**Table 4:** Mean values for all paddocks, including entrance areas, of total N (TN, %), total C (TC, %), total P (TP, mg kg^−1^), ammonium-lactate soluble P “easily soluble P” (P-AL, mg kg^−1^), calcium cloride soluble P “leachable P” (P-CaCl_2_, mg kg^−1^) and degree of P saturation of the soil (DPS, %), at three depths, standard deviations in parentheses. Significant differences (*p* < 0.05) are indicated by letters

Depth cm	TN %	TC %	TP mg kg^−1^	P-AL mg kg^−1^	CaCl_2_-P mg kg^−1^	DPS %
0–10	0.24^a^ (0.05)	2.8^a^ (0.46)	1016^a^ (101)	18^a^ (8.0)	0.60^a^ (0.31)	31^a^ (12)
10–20	0.16^b^ (0.03)	1.8^b^ (0.49)	894^a^ (96)	13^a^ (7.2)	0.28^ab^ (0.14)	25^a^ (10)
20–30	0.12^b^ (0.04)	1.34^b^ (0.41)	879^a^ (88)	12^a^ (3.5)	0.17^b^ (0.08)	22^a^ (6.5)

### N and P concentrations in ditch water

**Fig. 4:**
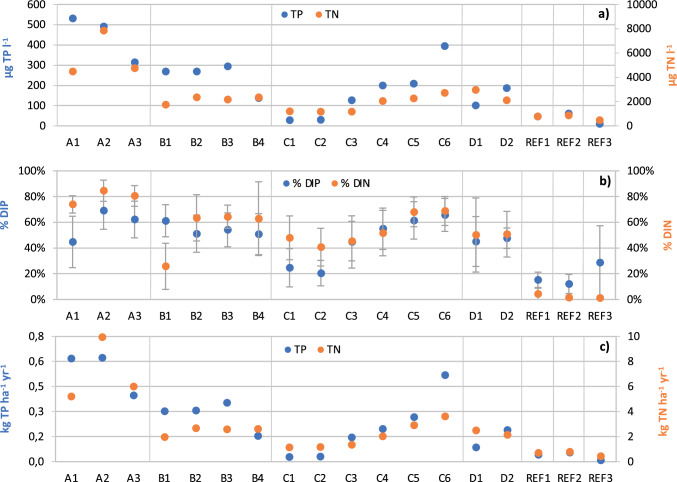
**a** Annual mean concentration (flow weighted) of total P and total N (µg TP l^−1^, µg TN l^−1^), **b** annual average of the relative amount of dissolved P (% DIP) and dissolved N (% DIN) of total P and N, and **c** area-normalized annual total P and N load (kg TP ha^−1^ year^−1^, kg TN ha^−1^ year^−1^) at the different sampling sites in catchment A–D and reference areas 1–3

The mean nutrient concentrations in the ditch water of the sub-catchments varied substantially, from 10 to 60 µg P l^−1^ and 500 to 900 µg N l^−1^ in the reference areas with 100% forest, to 500 µg P l^−1^ and nearly 8000 µg N l^−1^ in sub-catchments with horse farms surrounded by small agricultural fields, forest and open land including summer grazing areas (Fig. 4a). The mean concentrations of both P and N increased significantly with increasing proportion of paddocks in the landscape (Table 5). There were also significantly elevated P concentrations in sub-catchments with a high abundance of arable land and open land (Table 5). The lowest concentrations were found in the reference areas and sub-catchments with large portion of forest.

**Table 5:** The effect from surrounding landcover types on nutrient loadings from horse farms to sub-catchments in Ekerö municipality, Stockholm County, Sweden. All response variables (rows) were analysed with sub-catchment area, sampling location, nutrient load from private sewer and all landcover types as fixed factors and farm as random factor in linear mixed models. Columns report the result from the fixed landcover types and nutrient load from private sewers in the final most informative models following model selection. All effect values are estimated regression coefficients. Significant differences among sub-catchment areas and site locations are not reported. Landcover types lacking significant effect in all models are omitted

Variable	Paddock	Arable land	Open land	Forest	Sewers
Load (kg total P year^−1^)	1.52*	ns	1.90*	ns	0.77***
Load (kg total N year^−1^)	0.85°	ns	ns	ns	0.02**
Load per ha (kg total P ha^−1^ year^−1^)	0.63***	0.38*	0.63**	ns	ns
Load per ha (kg total N ha^−1^ year^−1^)	1.43**	0.75*	ns	ns	ns
Mean concentration (µg total P l^−1^)	442.90***	348.39***	457.16***	ns	ns
Mean concentration (µg total N l^−1^)	3942.48*	ns	ns	ns	ns
Dissolved P (% DIP)	0.58***	0.45***	0.43***	ns	ns
Dissolved N (% DIN)	ns	ns	ns	-0.4*	ns

ns *p* > 0.10, °*p* < 0.10, *p < 0.05, **p < 0.01, ***p < 0.001

### Proportion dissolved N and P

Of the total load of nutrients, the highest proportions of dissolved nutrients were found in sub-catchments where land use was dominated by paddocks, arable land and houses with private sewers (Fig. 4b, Table 5). The lowest proportions were found in the reference areas as well as in C1 and C2 (12–25% dissolved P, 1–48% dissolved N), where forest is the main land use, and where all houses were connected to the municipal water and sewage system (Fig. 4b). There was a significant positive effect from the proportions of paddocks, arable land and open land, on dissolved P, and a significant negative effect from the proportion forest cover on dissolved N (Table 5).

### Nutrient loads (measured) from paddocks and the studied sub-catchments

Nutrient loads from sub-catchments with a large proportion of paddocks, arable land and/or houses with private sewers had higher area-specific nutrient loads (≥ 0.15 kg P ha^−1^ year^−1^, ≥ 2.0 kg N ha^−1^ year^−1^) than the other sub-catchments (< 0.15 kg P ha^−1^ year^−1^, < 2.0 kg N ha^−1^ year^−1^) (Fig. 4c), with a significant positive effect of the proportions of paddocks, arable land and open land (Table 5). In sub-catchments with a higher estimated annual P load from private sewers, there were also a significantly higher P and N load in the ditch water (Table 5). The area-specific nutrient load for horse facilities varied between the sub-catchments. However, on average, the load was similar between the “measurement” and “calculation” approach for P (1.2 kg P ha^−1^ year^−1^), but not for N (7.6 kg N ha^−1^ year^−1^ and 16.7 kg N ha^−1^ year^−1^, respectively) (Table 6).

**Table 6:** Average (minimum–maximum) values of area-specific total N and P load (kg ha^−1^ year^−1^) and type-specific total N and P concentration (mg l^−1^) for horse facilities, derived from the “measurement” and the “calculation” method from seven sub-catchments in three catchments

	Area-specific load		Type-specific concentration	
	kg TP ha^−1^ year^−1^	kg TN ha^−1^ year^−1^	mg TP l^−1^	mg TN l^−1^
Measurement method	1.24 (0.4–3.2)	7.60 (3.1–22.3)	1.05 (0.4–2.7)	6.38 (2.7–18.9)
Calculation method	1.19 (0.8–1.7)	16.66 (11.3–23.2)	1.01 (0.7–1.5)	14.00 (10.9–20.2)

### N and P load and source distribution in the studied areas at the catchment/landscape level

**Fig. 5:**
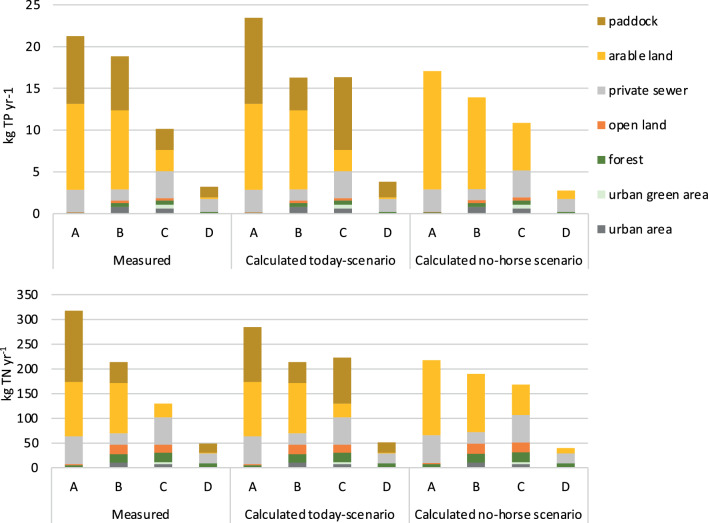
Estimated annual phosphorus and nitrogen load (kg TP year^−1^, kg TN year^−1^) in the four catchment areas; measured, calculated today-scenario (with horse activities) and calculated no-horse scenario (without horse activities). Source distribution for measured load was derived from standard values, site-specific runoff and proportion land use, for all nutrient sources but paddock, which was assumed to be the residual load. The calculated load from paddocks (today-scenario) assumes an area-specific load twice the load from arable land

The “measured” total annual nutrient loss from the studied sub-catchments to the recipient ranged from 5 to 20 kg P and from 50 to 320 kg N (Fig. 5), where the variability was due to size and land use. This corresponded to 0.15–0.40 kg P ha^−1^ year^−1^ and 1.6–6.1 kg N ha^−1^ year^−1^. The main nutrient sources were arable land, paddocks and sewage from private homes (Fig. 5). Forest and open land contributed less, and they often covered a large part of the sub-catchments.

In catchments A, B and D, there were less than 15% difference between the measured and the calculated loads, whereas for catchment C, the total load estimated from “measurements” was ca 40% lower than estimated from “calculations”.

Assessment at the catchment scale of the source distribution of the “measured” loads suggested that paddocks contributed with 30–40% of the annual P load and 20–45% of the N load in the A, B and D sub-catchments. This was similar to the difference between the calculated “today-scenario” and “no-horse-scenario”, suggesting that the presence of horse facilities increased the nutrient load with approximately 15–30% P and 10–25% N. The lowest increase (10–15%) was found in sub-catchment B. This sub-catchment is very large and has a fairly small proportion of paddocks and a large proportion of fields. Arable fields contribute with large nutrient loads, resulting in a relatively small impact from horse facilities on the total nutrient load.

## Discussion

Eutrophication of inland and coastal waters is the result of multiple nutrient sources (HELCOM 2018a, b), and land use is an important factor affecting the nutrient loss and water quality in the catchments (e.g. Hong et al. 2012; Kändler et al. 2017; de Wit et al. 2020; Djodjic et al. 2021). To reach the environmental goals of no eutrophication, there is a need to assess the nutrient loads at different scale levels, and provide means for local landowners to actively participate in mitigation measures. This study was performed in a multi-functional peri-urban landscape with a mixture of land uses and anthropogenic activities, such as farming, equine facilities, recreational trekking areas, nature reserves and residential areas with private sewers, gardens and roads. Despite the complexity of patchy landscapes, our results showed that horse activities can enhance the nutrient transport considerably and should be included in eutrophication assessments, especially for horse-dense peri-urban areas. The nutrient concentrations increased in the water downstream of the paddocks and the proportion of dissolved nutrients increased with increased proportion of land used for paddocks and grazing areas; soil sampling in paddocks confirmed a risk of P losses from these areas due to accumulation of P in the soil. The amounts of soluble P, directly available for leaching, roughly correspond to be 0.6–1.1 kg ha^−1^ (if soil density equals 1300 kg m^−3^), which is considerably higher than the arable field (0.2 kg ha^−1^). Further, also bridleways and summer grazing areas should be considered in assessment of the effects of horses on water quality, as horses, in contrast to livestock, move around in the landscape outside fenced areas. Site visits revealed horse manure on tracks and walkways, which indicate that horse activities possibly have contributed to the observed effects.

### Assessing local nutrient loads in complex, multi-functional catchments

Significant influence of land use pattern on water quality has also been found in other studies, where factors for increased impact included livestock farming and arable land, densely populated areas and horse farming (e.g. Woli et al. 2004; Kändler et al. 2017; Vrebos et al. 2017; de Wit et al. 2020; Djodjic et al. 2021). Landscape composition (proportion of land uses) and configuration (spatial arrangement) are also important factors for nutrient transport (Casquin et al. 2021).

However, the impact of horses on eutrophication seems often to be overlooked or underestimated, probably due to the fact that nutrient losses from diffuse nutrient sources are difficult to assess and requires considerable resources. Further, as horse-keeping for recreation is a relatively new and growing sector, compared to agriculture, there has been a lack of awareness and regulations directed at horse facilities, where manure removal and recirculation can be a considerable cost (Hammer et al. 2017).

Nutrient loads from horse facilities in our study (1.2 kg P ha^−1^ year^−1^ and 7.6 kg N ha^−1^ year^−1^) were based on field measurements during 1 year in four catchments with horses, combined with site-specific modelling of the water runoff (Table 6). These values are similar for N but higher for P, compared to values from long-term measurements in a catchment in the same part of Sweden (0.5 kg P ha^−1^ year^−1^ and 6.3 kg N ha^−1^ year^−1^) that is dominated by arable land, and have few houses and no livestock or horses (Linefur et al. 2022). Poor manure management can be one explanation for the higher P load from areas with horse facilities, and a plausible explanation for why this was not accompanied with increased N load could be volatile losses of N. Ammonia losses from urine, which contains the main part of excreted N in the form of ammonium, are supposed to be considerable when placed on the soil surface, especially under conditions with summer temperatures (Sommer and Hutchings 2001). Moreover, N losses by denitrification will occur from systems where nitrate is enriched (Fowler et al. 2013). A study of horse paddocks without vegetation in comparison with hay field and grassland showed that larger amounts of nitrate in the paddock soil was followed by increased losses of nitrous oxides (Makinde 2020).

The four catchments in the study represented different mixes of land use, from mainly rural open land to forest-dominated and mixed land use, including more urban and residential areas. To assess nutrient loads and target suitable mitigation measures, we applied and compared two approaches, one mainly relying on field measurements with one based on calculations using standard values. Overall, the two approaches gave similar results for P but not for N, suggesting that the assumption for the calculation approach that horses contribute “two times arable land” was valid for P, but overestimated for N. Further, the methods produced similar results for three of the four catchments, both at the catchment and at the sub-catchment levels, implying that both methods are applicable for this type of assessments.

At the catchment levels, the difference of the total nutrient loads between the two approaches was less than 15% for three of the four catchments. The largest discrepancy (40%) was found for the patchiest catchment (C), possibly due to the limitation of standard values to handle high land use complexity. Catchment C has one sub-catchment with numerous single-family homes and blockhouses, two sub-catchments with quite a few detached houses with private sewers, and walk ways and open areas for recreational use that runs through several sub-catchments. Also, substantial ditch cleaning took place in one of the sub-catchments, possibly affecting the outcome. The contribution from paddocks in catchment C was likely underestimated, especially for N. Nearly 20% of the land was covered by paddocks and several significant effects from paddocks were detected (Table 5).

In addition to challenges related to handling patchiness and land use complexity in eutrophication assessments, there are also other considerations regarding the suitability of these methods as a basis for mitigation and management. It is costly and resource demanding to quantify nutrient loads from diffuse nutrient sources with field measurements, and is often associated with uncertainties and a natural variability to consider. The catchment areas for representative sampling sites need to be identified and the water runoff was measured or modelled. Flow proportional water sampling is often preferable in order to improve the accuracy of the estimations (Schleppi et al. 2006), but not always possible. Using standard values for runoff for the region instead of the runoff for each sub-catchment derived from the site-specific water flow model resulted in ca 30–40% higher N and P loads in this study, stressing the importance of adequate estimates of the water runoff. In addition to geographic scale, the time scale is also important. Seasonal and annual weather variations cause large variations in nutrient transport in waterways, especially due to variations in precipitation and water runoff (Ezzati et al. 2023). The year of our study was drier than normal with 30% lower precipitation than the average for 1981–2021 (693 mm year^−1^). This most probably resulted in lower runoff compared to an average year and probably also in lower total transport of nutrients due to less efficient outwashing of water-soluble N and P during drier conditions.

Using models based on area-specific coefficients is relatively simple, straight forward, and a commonly used approach (Metson et al. 2017; HELCOM 2022) and is considered to be a practical decision support tool for assessing the impacts of land use on water quality (Palviainen et al. 2016). However, using area-specific load coefficients introduce uncertainties, and it is important to select representative values of both source-specific coefficients and water runoff. This comparative study suggests that assessments using standard values can provide a fair overview of nutrient sources and losses for the catchment level, especially in combination with high physical presence and local knowledge to gain a better understanding of landscape under assessment.

Another finding in the study concerns the impact of private sewers on nutrient load in the catchments. Although there were relatively few houses in all the studied catchments, we found significant effects of private sewers on the nutrient load. Just as horses, humans have high proportions of dissolved N in urine and P in feces (Mihelcic et al. 2011; Ögren et al. 2013; Weir et al. 2017), and most of the households relied on infiltration beds or other ground-based sewage solution systems in which reduction efficiencies may vary. Consequently, in addition to horses, private sewers are important to consider in nutrient load assessments, especially in small patchy catchments.

In the reference areas of the study sites, all dominated by forest, the nutrient concentrations were higher than expected, possibly due to the influence of nutrient sources and activities such as bridleways, various outdoor recreation activities and walking of dogs. Such sources contribute to composite water samples and override the low nutrient concentrations from the forests.

### Risk factors for nutrient loads—assessments across multiple scales

The nutrient concentrations in the ditch water varied considerably over the study period. Increased amounts of soluble P in soil surface layers in paddocks were identified, which also constitute a risk to enhanced losses of dissolved and particle-bound P, both through subsurface leaching and surface runoff. Erosion mainly occurs when there is poor vegetation and when infiltration of water is reduced, during wet conditions or snow melting periods (Norberg et al. 2022).

**Fig. 6:**
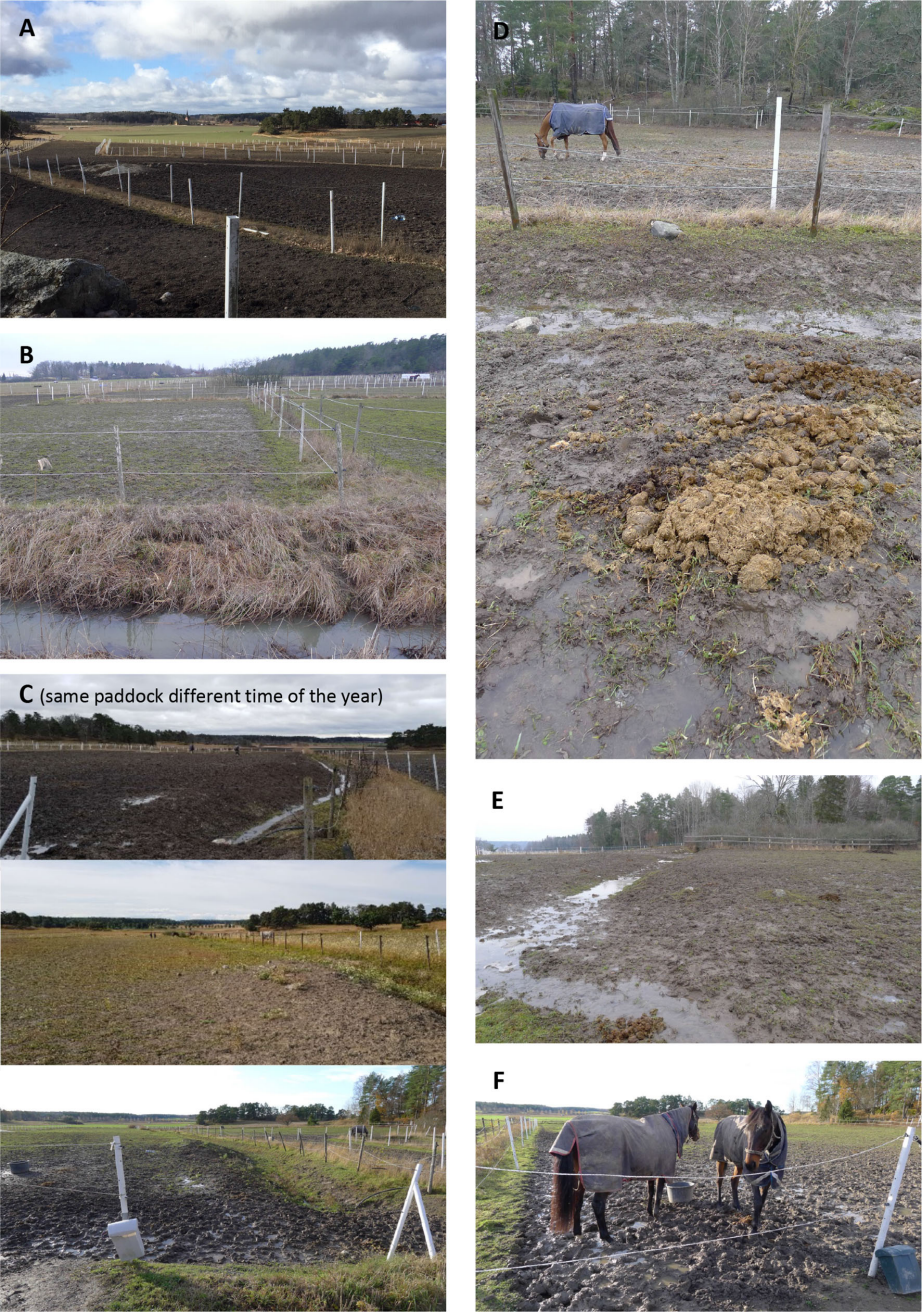
Risk factors for nutrient losses in horse paddocks. **A**–**F** Trampled grounds reduce the nutrient retention. **C**–**E** Manure piles left in the paddocks, poor drainage and ditches running through paddocks. **B** Nutrients from paddocks located next to ditches can readily be transported to lakes or coastal areas. **F** The vegetation cover lost due to trampling by horses, is often worst at entrances and feeding spots. (Photos by L. Kumblad)

To investigate the reasons and identify the potential risk factors for enhanced nutrient transport, the elevated values were cross-checked for possible explanations in field observations during sampling. The most elevated values could be directly related to the presence of horses. The ground cover in most paddocks used for daily turn-out was heavily affected by horse trampling and lacked rooted vegetation on substantial parts most of the year (Fig. 6). Conditions were worst during the wet period in November–April, with open mud, puddles and no vegetation. In May–October, conditions were drier and some parts of the paddocks were covered with poor and patchy vegetation. At the entrance of the paddocks, the ground was vegetation free the whole year, and there was also high manure load throughout the period in these areas, which also was in accordance with the results from the soil sampling (Fig. 3). Enclosures that were used only for summer grazing with a low density of horses had a constant vegetation cover. The most evident risk factor observed during sampling was trampling in the vicinity of ditches (sampling site), especially during or just after rain, as well as during snow melting periods. Other non-horse related reasons for elevated nutrient transport were ditch cleaning, establishments of new ditches and overflooding wells of bio-treatment sewage plants.

The high value and competition of land close to cities can result in persistent high densities of horses in the paddocks. As a result, these paddocks often lose their vegetation cover that can prevent erosion and trap nutrients. Risk factors affecting the potential for nutrient leaching could be identified on different scales: locally in the paddocks or in the vicinity, on the horse farm, sub-catchment and landscape levels. Closeness to water courses as well as how the paddock and horse farm are placed in the landscape can affect the concentrations in ditch water. This stresses the need for frequent manure removal as shown by, e.g. Aronsson et al. (2022) and the need for measures to reduce trampling damages in paddocks in the strive for an intact vegetation cover. Also, composted horse manure can be used as a resource for improved soil fertility in crop production (Bernal et al. 2009). Hence, there are a range of important and practical management measures that belong to the local farm level that can improve the water quality at the catchment level.

### From horse farm to landscape—assessments across multiple scales

Analyses of anthropogenic nutrient fluxes for the Baltic Sea catchment show a strong linear relationship between the anthropogenic nutrient input and riverine nutrient fluxes, and compliance to the Baltic Sea Action Plan (HELCOM 2021) would imply substantial changes in the agricultural sector (Hong et al. 2012, 2017; McCrackin et al. 2018a). As horse facilities were not included in the Baltic Sea nutrient accounting analyses, and result from this study clearly shows that horses may contribute considerably in some areas, the net anthropogenic nutrient input to the region is possibly underestimated. Comparisons of the average annual P and N loads from horses (ca 0.5 g P m^−2^, 3 g N m^−2^) and humans (ca 0.005 g P m^−2^, 0.1 g N m^−2^) in the studied catchments in this study show a ca 100 times higher P load and 40 times higher N load from horses than from humans. The calculations were based on the number of horses and humans living in the respective catchments, excretion values for sport horses (Malgeryd and Persson 2013) and nutrient content in toilet wastewater for humans (Jönsson et al. 2005), with reduction due to sewage treatment (Palm et al. 2002 and Hübinette 2009). Horse farms in peri-urban areas can thus be considered as nutrient hot-spots, as most of the fodder is imported to the catchment, the manure is seldom recycled within the catchment, and intense trampling contributes to increased nutrient losses from the soil. To prevent further accumulation and losses of nutrients, the recycling of manure and human sewage need to be more efficient (McCrackin et al. 2018a; Svanbäck et al. 2019; Pihlainen et al. 2020), and to enhance the efficiency in environmental and economic outcomes, abatement work should focus on the dissolved and thus biologically available P (Iho et al. 2023).

Mitigation measures to decrease nutrient loads need to be viewed in an ecosystem management perspective in line with the Water Framework Directive approach, integrating stakeholders and managers at different scale levels from the local field and farm to the catchment and river basins (EC 2000; Hammer et al. 2011). Many horse facilities in Sweden and other western countries are situated in the peri-urban landscape with a matrix of different land use that contribute with nutrients to the adjacent waters to various extents (Elgåker 2012). At the catchment level in our study, it was shown that a higher proportion of paddocks increased the mean nutrient concentrations in ditch water, the amount of dissolved nutrients and the load of P and N from the particular area. The present study also illustrates the need to study nutrient loads at different scales, where horse facilities, the proportion of arable land and open land and the number of sewers from private households all contribute to the nutrient load. Local knowledge is vital to be able to understand the surroundings of the horse facilities, manure practices and the location of paddocks within the catchment and to elucidate different risk factors that can affect the nutrient load, and if needed, can be managed. For individual horse-keepers to be willing to take actions to decrease the risk for nutrient leaching, solid estimates of the actual contributions to nutrient load are needed (Franzén et al. 2016).

## Conclusion

Horse-keeping facilities can contribute considerably to the nutrient load to surface waters, particularly in multi-functional peri-urban landscapes. Local risk factors include manure management, trampling severity, soil condition, closeness to water and where the facility is situated in the landscape. Adequate methods are needed to distinguish the impact from horses, from the impact of other nutrient sources. In small, patchy and complex areas, measurements from representative sampling sites are needed in combination with site-specific water runoff estimates, and local knowledge or site visits. Literature standard values may produce good estimates at the larger catchment level. The impact of relatively small changes in the landscape, such as expansions of paddocks, new residence houses or ditch cleaning, may have a profound influence of the overall nutrient transport at the local scale. Catchment-based management should include all local sources, both in monitoring and assessment programmes. The assessments need to be applied at multiple scale levels, but adapted to local conditions. To reduce the impact from horse-keeping, guidelines and incentives for manure management that enables a sustainable recirculation of nutrients need to be improved and developed. Local engagement and commitment are needed not only to identify and quantify the problems, but also to find the appropriate solutions.

## Data Availability

Altitude—Lantmäteriet (FUK licence). Air photo—Lantmäteriet (FUK licence). Landcover—Swedish Environmental Protection Agency (open data). Meteorological and hydrological data—temperature, precipitation, SVAR_2016_3—SMHI (open data). Soil—Swedish Geological Survey (SGU) (FUK licence).
